# Cross-sectional study on the association between neutrophil-percentage-to-albumin ratio (NPAR) and prevalence of stroke among US adults: NHANES 1999–2018

**DOI:** 10.3389/fneur.2025.1520298

**Published:** 2025-01-28

**Authors:** Chenglin Ye, Yong Mo, Tiansheng Su, Guangxiang Huang, Jiachao Lu, Shuling Tang, Qianrong Huang, Qiuyun Li, Qian Jiang, Fangzhou Guo, Pinghua Wu, Guozhong Zhang, Jun Yan

**Affiliations:** ^1^Department of Neurosurgery, Guangxi Medical University Cancer Hospital, Nanning, China; ^2^Department of Breast Surgery, Guangxi Medical University Cancer Hospital, Nanning, China; ^3^Department of Neurosurgery, Institute of Brain Diseases, Nanfang Hospital of Southern Medical University, Guangzhou, China

**Keywords:** NPAR, stroke, NHANES, a cross-sectional study, restricted cubic

## Abstract

The neutrophil-to-albumin ratio (NPAR) is a relatively novel composite biomarker of inflammation, which has been used for prognostication in cardiovascular diseases and may also be associated with stroke. A cross-sectional analysis was conducted using data from the National Health and Nutrition Examination Survey (NHANES) 1999–2018, including 48,734 individuals with complete NPAR and stroke data. The association between stroke prevalence and NPAR values was assessed through multivariate regression analysis. The relationship between these variables was further visualized using restricted cubic splines (RCS). Additionally, potential factors influencing this relationship were explored through subgroup analysis. The regression model revealed a significant association between NPAR and stroke prevalence, even after adjusting for other covariates [1.06 (1.04, 1.08)]. Stroke prevalence was 62% higher in the highest NPAR group compared to the lowest [1.62 (1.40, 1.89)]. The RCS analysis further confirmed this positive correlation. Subgroup analysis showed that this association was not significantly influenced by other factors. This study establishes a strong association between NPAR and stroke prevalence. However, further studies are needed to clarify the underlying mechanisms and establish a direct causal link.

## 1 Introduction

Cerebrovascular disorders, such as stroke, are characterized by the blockage of blood vessels (1). SStroke is the third leading cause of mortality and disability globally and the second leading cause of death worldwide. Between 1990 and 2019, stroke incidence increased by 70%, and its global prevalence rose by 85% (2). This growing trend is largely attributable to the aging population (3). Stroke remains a major public health challenge, as it is responsible for high rates of morbidity, mortality, and disability. Moreover, the global annual economic burden of stroke exceeds $89.1 billion, placing a significant strain on healthcare systems (4). Moreover, the global annual economic burden of stroke exceeds $89.1 billion, placing a significant strain on healthcare systems (5). Identifying and managing controllable and preventable risk factors is therefore crucial in reducing stroke incidence. The development of novel biomarkers facilitates early detection of stroke risk (6, 7), thereby helping to lower stroke rates within the population.

The pathogenesis of stroke is closely linked to inflammation, with acute ischemic stroke involving endothelial activation, blood–brain barrier disruption, the release of inflammatory mediators, oxidants, and cytokines, and infiltration by platelets and leukocytes (8, 9). Inflammation is one of the first responses to cerebral injury following a stroke, and it also plays a key role in tissue repair during the recovery phase (10). Evaluating peripheral leukocytes, particularly neutrophils, offers a cost-effective and accessible approach to assess inflammation (11). Neutrophil activation in stroke-damaged brain tissue attracts immune cells, which in turn produce a variety of substances that exacerbate inflammation (12).

Albumin, a protein constituting over half of the serum’s protein content, is an intermediate-sized protein with a molecular weight ranging between 66 and 69 kDa (13). It performs essential roles in osmoregulation, antioxidation, and anti-inflammatory processes (14). NPAR, which combines both neutrophil percentage and albumin levels, is an emerging biomarker used to assess immune and systemic inflammatory conditions (15). Reduced albumin levels have been strongly associated with poor outcomes in stroke patients (16). Previous studies have shown that elevated neutrophil counts in the early stages of stroke are associated with greater stroke severity (17). NPAR is increasingly utilized in research on disease risk and outcomes. For example, myocardial infarction patients with elevated NPAR have been found to have higher hospital mortality (18). A prior retrospective study indicated that NPAR is independently associated with stroke recurrence within 3 months following the first acute stroke, suggesting that NPAR might be a better predictor of acute ischemic stroke recurrence than albumin levels or neutrophil percentage alone (19). A recent investigation further revealed that NPAR was associated with ICU hospitalization in ischemic stroke patients (20). Collectively, these findings suggest a potential link between NPAR and stroke. However, the existing data on this relationship remain limited, highlighting the need for more comprehensive research. This study aims to investigate the association between NPAR and stroke within the National Health and Nutrition Examination Survey (NHANES) cohort.

## 2 Materials and methods

### 2.1 Survey description

Data for this study were collected through the NHANES, conducted across the United States. The survey employed a stratified multistage random sampling design, which provided a robust framework for assessing the health and nutritional status of the U.S. population (21). Participants underwent a range of assessments, including laboratory tests, home interviews, physical examinations, and additional evaluations. Written informed consent was obtained from all participants prior to their inclusion in the study (22). NHANES, with its extensive database, serves as a comprehensive and reliable tool for deriving population-level insights.

### 2.2 Study population

**Figure 1:**
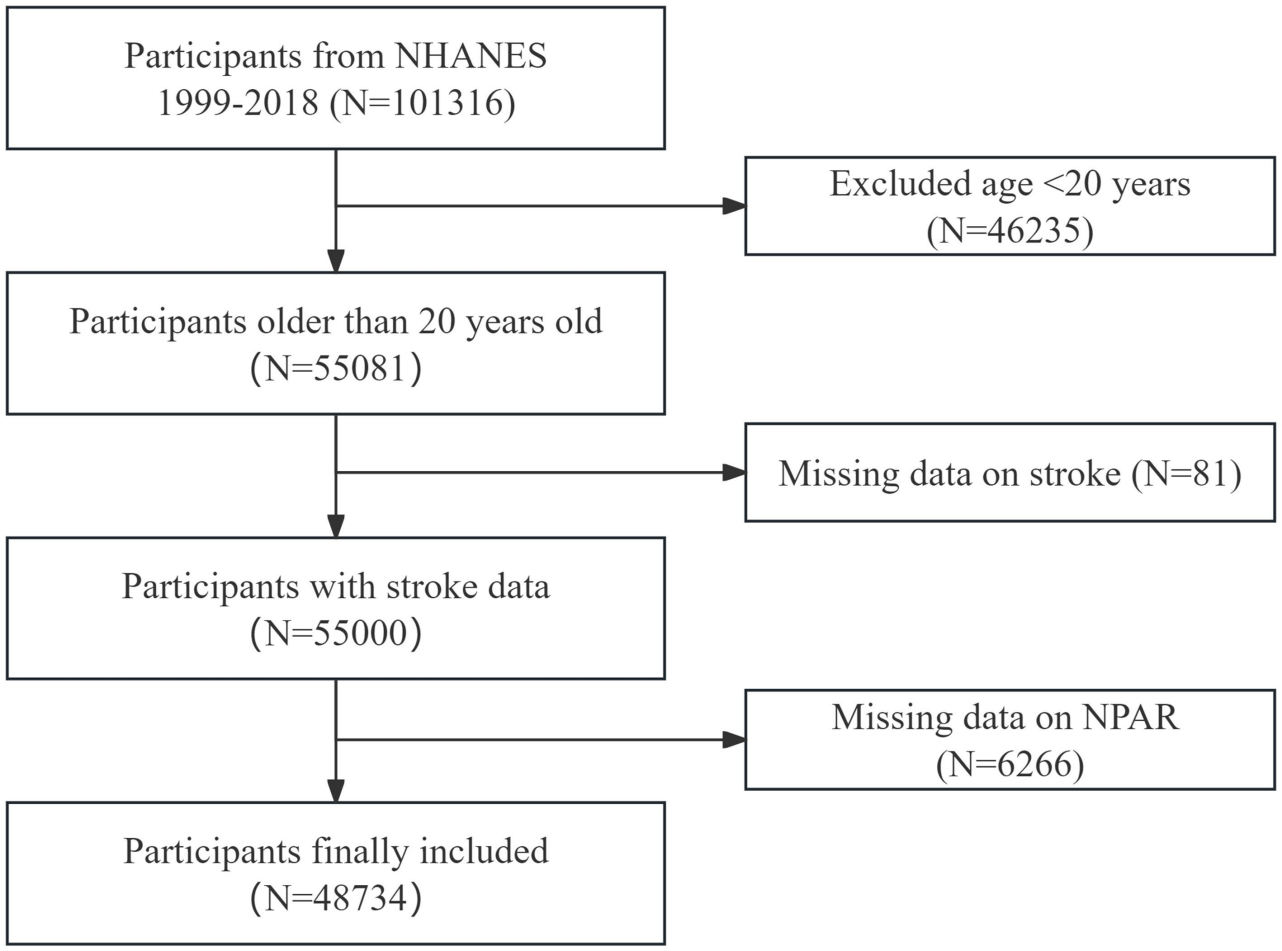
Flowchart of participant selection. This flowchart illustrates the process used to select participants for the study, from initial screening to final inclusion. The figure shows the number of participants excluded at each stage and the final cohort included in the analysis.

Data from 10 cycles of NHANES, spanning from 1999 to 2018, were analyzed in this study, providing broad population coverage and a large sample size. After applying the inclusion and exclusion criteria, 48,734 individuals were enrolled (Figure 1). The exclusion criteria were as follows: (1) individuals under 20 years of age, (2) those with incomplete stroke data, and (3) participants missing NPAR data.

### 2.3 Assessment of NPAR

The Coulter VCS system was used to determine the white blood cell (WBC) differential. NPAR was calculated using the following formula: (Percentage of neutrophils among total white blood cell count) × 100 ÷ Albumin (g/dL) (23). The same blood sample was used for both the WBC differential and albumin measurement. NPAR values were used as the primary exposure variable in this study.

### 2.4 Diagnosis of stroke

To assess stroke occurrence, a medical conditions questionnaire was administered. Participants were classified as having experienced a stroke if they answered “yes” to the question: “Has a doctor or other health professional ever informed you that you had a stroke?” Previous studies have demonstrated the reliability of self-reported stroke data (24). In this study, stroke was considered the primary outcome variable.

### 2.5 Data processing for covariates

Based on existing literature, several factors related to stroke and NPAR were identified as covariates. These included age, gender, ethnicity, educational level, poverty-to-income ratio (PIR), physical activity (PA), body mass index (BMI), tobacco and alcohol consumption, hypertension, and diabetes status. Upon examining the NHANES dataset, we noted the presence of missing data for several key variables. Multiple imputation (MI) was employed to handle the missing data.

The rationale for selecting MI over alternative methods, such as full case analysis or single imputation, is multifaceted. MI preserves the inherent variability and structure of the dataset by generating multiple plausible datasets, where missing values are estimated based on observed data. This approach mitigates the bias inherent in single imputation methods, which can underestimate variability and lead to overly confident results. By incorporating uncertainty from the missing data, MI produces more reliable and efficient estimates and standard errors, offering more accurate statistical conclusions compared to methods that fail to address this uncertainty adequately. Furthermore, MI can improve model performance by accounting for missing data-related uncertainty, thereby enhancing the accuracy and precision of parameter estimates. This contributes to more dependable findings and predictions. MI also helps stabilize estimates, reducing potential bias caused by missing data, which in turn improves the consistency and generalizability of the results.

### 2.6 Statistical analysis

All statistical analyses were conducted using R and EmpowerStats software. Participants’ characteristics were compared across NPAR quartiles using chi-square tests for categorical variables and t-tests for continuous variables. Multivariate logistic regression was employed in three separate models to explore the linear relationship between NPAR and stroke. These models were as follows: Model 1: No covariates were adjusted. Model 2: Adjustments were made for age, gender, ethnicity, and education. Model 3: A broader set of covariates was included, such as age, gender, ethnicity, education, alcohol consumption, smoking, diabetes, hypertension, PA, and PIR. After converting NPAR scores into quartiles, trend tests were performed to evaluate the linear association between NPAR and stroke prevalence. Subgroup analyses were then conducted to examine this relationship further, using various stratifying factors. Additionally, RCS analysis was performed to assess the potential for nonlinear relationships between NPAR and stroke occurrence.

## 3 Results

### 3.1 Baseline characteristics of participants

The study included 48,734 participants with a mean age of 46.93 years (SD = 16.90). Of the participants, 48.24% were male and 51.76% were female. The overall stroke prevalence was 3.76%, with rates increasing progressively across higher NPAR quartiles. The baseline characteristics of the participants are summarized in Table 1, which organizes the data by NPAR quartiles. The mean NPAR for the entire cohort was 13.93 ± 2.86, with quartile cutoffs as follows: Quartile 1: <12.11; Quartile 2: 12.11–13.76; Quartile 3: 13.76–15.48; Quartile 4: >15.48. Participants in the higher NPAR quartiles were more likely to be older, predominantly female, have lower educational attainment, and have a higher prevalence of smoking. They also exhibited a greater prevalence of diabetes, hypertension, and stroke compared to the general population. Additionally, higher NPAR values were associated with increased levels of BMI, waist circumference (WC), body weight, triglycerides, LDL-C, and total cholesterol (TC), while PIR and HDL-C levels were relatively lower. This pattern suggests a possible link between unhealthy lifestyle choices and elevated NPAR levels. Further analysis of stroke prevalence across various demographics (Table 2). The findings indicated that older age, female gender, obesity, low educational attainment, poverty, smoking, hypertension, and diabetes were associated with a greater prevalence of stroke. Stroke patients have elevated levels of CRP, FPG, HbA1c, TG, and NPAR.

**Table 1:** Baseline characteristics according to NPAR quartiles.

Characteristics	Total (*N* = 48,734)	Quartiles of NPAR				
		Q1 (<12.11)	Q2(12.11–13.76)	Q3(13.76–15.48) (2.54–3.43)	Q4 (> 15.48)	*p* value
Age (years)	46.93 ± 16.90	43.76 ± 16.30	45.61 ± 16.13	48.22 ± 16.58	50.41 ± 17.94	<0.0001
Gender (%)						<0.0001
Male	48.24	57.61	51.9	45.98	36.5	
Female	51.76	42.39	48.1	54.02	63.5	
Race (%)						<0.0001
Mexican American	8.19	7.69	8.72	8.47	7.79	
Other Hispanic	5.67	5.75	6.17	5.46	5.22	
Non-Hispanic White	68.62	62.22	69.19	71.51	71.58	
Non-Hispanic Black	10.65	16.4	8.83	8.13	9.41	
Other	6.88	7.94	7.09	6.43	6	
Education (%)						<0.0001
Less than high school	17.23	17.14	16.56	16.95	18.39	
High school grade	23.97	22.4	23.88	24.42	25.28	
Some college or above	58.8	60.46	59.57	58.63	56.33	
PIR (%)						<0.0001
Low	21.25	20.82	19.95	20.67	23.87	
Moderate	36.03	35.47	35.31	35.56	37.97	
High	42.72	43.71	44.74	43.77	38.16	
Drinking status (%)						<0.0001
Never	19.06	17.55	17.88	18.91	22.34	
Moderate	39.59	39.64	40.59	40.01	37.83	
Heavy	41.36	42.81	41.53	41.08	39.83	
Smoking Status (%)						<0.0001
No	53.8	55.39	55.13	53.34	51.06	
Yes	46.2	44.61	44.87	46.66	48.94	
PA level (%)						<0.0001
Low	26.35	22.78	23.25	27.36	32.62	
Moderate	38.37	36.61	38.21	38.04	40.81	
High	35.28	40.61	38.54	34.6	26.57	
Hypertension (%)						<0.0001
No	62.38	67.76	65.46	60.99	54.59	
Yes	37.62	32.24	34.54	39.01	45.41	
Diabetes (%)						<0.0001
No	87.82	91.58	90.44	87.31	81.35	
Yes	12.18	8.42	9.56	12.69	18.65	
BMI (kg/m^2^)	28.76 ± 6.72	27.30 ± 5.58	28.12 ± 5.99	29.07 ± 6.58	30.75 ± 8.15	<0.0001
CRP (mg/L)	0.41 ± 0.80	0.24 ± 0.41	0.29 ± 0.49	0.39 ± 0.64	0.78 ± 1.31	<0.0001
WAIST (cm)	98.33 ± 16.26	94.66 ± 14.44	96.96 ± 15.18	99.35 ± 16.21	102.87 ± 18.18	<0.0001
FPG (mg/dL)	105.23 ± 31.11	101.15 ± 23.31	103.40 ± 26.84	106.50 ± 33.41	110.28 ± 38.59	<0.0001
HbA1c (%)	5.57 ± 0.91	5.48 ± 0.79	5.51 ± 0.83	5.59 ± 0.92	5.71 ± 1.09	<0.0001
HDL (mg/dL)	53.19 ± 16.26	53.76 ± 16.73	52.98 ± 16.07	52.77 ± 15.93	53.30 ± 16.29	<0.0001
LDL (mg/dL)	115.69 ± 35.38	117.53 ± 36.28	117.38 ± 34.81	117.30 ± 35.07	110.15 ± 34.79	<0.0001
TC (mg/dL)	196.06 ± 41.32	197.79 ± 42.30	197.87 ± 40.87	196.33 ± 40.32	191.82 ± 41.56	<0.0001
TG (mg/dL)	131.5 ± 103.95	125.8 ± 107.49	131.79 ± 103.62	134.48 ± 100.53	134.23 ± 103.81	<0.0001

**Table 2:** Baseline characteristics according to stroke.

Characteristics	Total (*N* = 48,734)	Non-stroke (*N* = 46,904)	Stroke (*N* = 1830)	*p* value
Age (years)	46.93 ± 16.90	46.43 ± 16.70	64.69 ± 14.36	<0.0001
Gender (%)				<0.0001
Male	48.24	48.40	42.67	
Female	51.76	51.60	57.33	
Race (%)				<0.0001
Mexican American	8.19	8.30	4.28	
Other Hispanic	5.67	5.73	3.22	
Non-Hispanic White	68.62	68.53	71.65	
Non-Hispanic Black	10.65	10.56	13.90	
Other	6.88	6.88	6.95	
Education (%)				<0.0001
Less than high school	17.23	16.90	28.84	
High school grade	23.97	23.82	29.42	
Some college or above	58.8	59.28	41.74	
PIR (%)				<0.0001
Low	21.25	20.94	32.21	
Moderate	36.03	35.80	43.93	
High	42.72	43.25	23.86	
Drinking status (%)				<0.0001
Never	19.06	18.67	35.83	
Moderate	39.59	39.58	39.87	
Heavy	41.36	41.75	24.30	
Smoking Status (%)				<0.0001
No	53.8	54.14	41.81	
Yes	46.2	45.86	58.19	
PA level (%)				<0.0001
Low	26.35	25.71	49.05	
Moderate	38.37	38.49	34.07	
High	35.28	35.80	16.88	
Hypertension (%)				<0.0001
No	62.38	63.58	20.51	
Yes	37.62	36.42	79.49	
Diabetes (%)				<0.0001
No	87.82	88.45	65.47	
Yes	12.18	11.55	34.53	
BMI(kg/m^2^)	28.76 ± 6.72	28.73 ± 6.71	29.90 ± 6.88	<0.0001
CRP(mg/L)	0.41 ± 0.80	0.41 ± 0.79	0.65 ± 1.10	<0.0001
WAIST(cm)	98.33 ± 16.26	98.20 ± 16.25	103.50 ± 16.03	<0.0001
FPG(mg/dL)	105.23 ± 31.11	104.84 ± 30.56	118.64 ± 43.96	<0.0001
HbA1c (%)	5.57 ± 0.91	5.56 ± 0.90	6.05 ± 1.27	<0.0001
HDL(mg/dL)	53.19 ± 16.26	53.24 ± 16.24	51.70 ± 16.61	0.0007
LDL(mg/dL)	115.69 ± 35.38	115.89 ± 35.20	108.87 ± 40.60	<0.0001
TC(mg/dL)	196.06 ± 41.32	196.23 ± 41.16	189.96 ± 46.07	<0.0001
TG(mg/dL)	131.5 ± 103.95	131.20 ± 104.21	143.38 ± 94.25	<0.0001
NPAR	13.82 ± 2.63	13.79 ± 2.62	14.79 ± 2.79	<0.0001

### 3.2 Association between the NPAR and stroke

**Figure 2:**
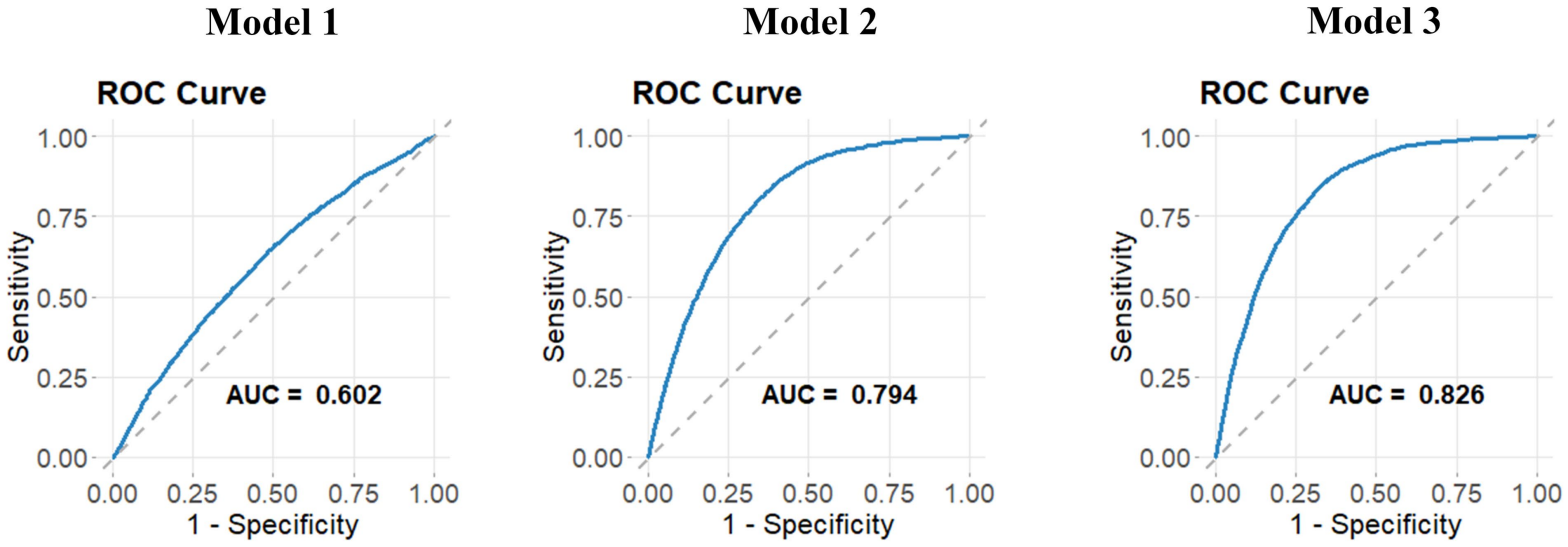
ROC curves for three models. ROC curves for three models are presented to assess their ability to discriminate between different outcomes. The curves plot sensitivity against 1-specificity, demonstrating the performance of each model in classifying stroke prevalence. Higher AUC values indicate better model performance in distinguishing stroke cases.

The relationship between NPAR and stroke incidence is presented in Table 3. In both the unadjusted model [OR 1.11 (1.09, 1.12)] and the partially adjusted model [OR 1.08 (1.07, 1.10)], a significant positive association was observed between NPAR and stroke. After applying full adjustments, the association remained statistically significant, with each unit increase in NPAR corresponding to a 6% increase in stroke prevalence [OR 1.06 (1.04, 1.08)]. The correlation remained robust even when NPAR was divided into quartiles, with stroke prevalence 62% higher in the highest NPAR quartile compared to the lowest. In comparison to the previous models, Model 3 showed lower Akaike Information Criterion (AIC), Bayesian Information Criterion (BIC), and pseudo-R^2^ values. Furthermore, the Receiver Operating Characteristic (ROC) curve analysis indicated that Model 3 had the highest area under the curve (AUC) value (Figure 2), with an AUC of 0.826, demonstrating excellent model performance.

**Table 3:** Association of NPAR with stroke.

Exposure	OR (95%CI), *p* value		
	Model 1^a^	Model 2^b^	Model 3^c^
NPAR	1.11 (1.09, 1.12) <0.0001	1.08 (1.07, 1.10) <0.0001	1.06 (1.04, 1.08) <0.0001
NPAR quartile			
Q1 (<12.11)	1	1	1
Q2 (12.11–13.76)	1.30 (1.11, 1.52) 0.0011	1.29 (1.10, 1.52) 0.0019	1.26 (1.07, 1.48) 0.0063
Q3 (13.76–15.48)	1.80 (1.55, 2.09) <0.0001	1.54 (1.32, 1.79) <0.0001	1.44 (1.23, 1.68) <0.0001
Q4 (> 15.48)	2.55 (2.21, 2.94) <0.0001	1.89 (1.63, 2.19) <0.0001	1.62 (1.40, 1.89) <0.0001
AIC	12105.22	10578.38	10106.73
BIC	12123.38	10631.63	10225.08
Pseudo R^2^	0.013	0.139	0.179
AUC	0.602	0.794	0.826

a No-adjusted model: adjusted for none.

b Minimally adjusted model: adjusted for age, gender, race, and education.

c Fully adjusted model: adjusted for age, gender, race, education, PIR, BMI, HbA1c, smoking status, drinking status, physical activity, hypertension, and diabetes.

**Figure 3:**
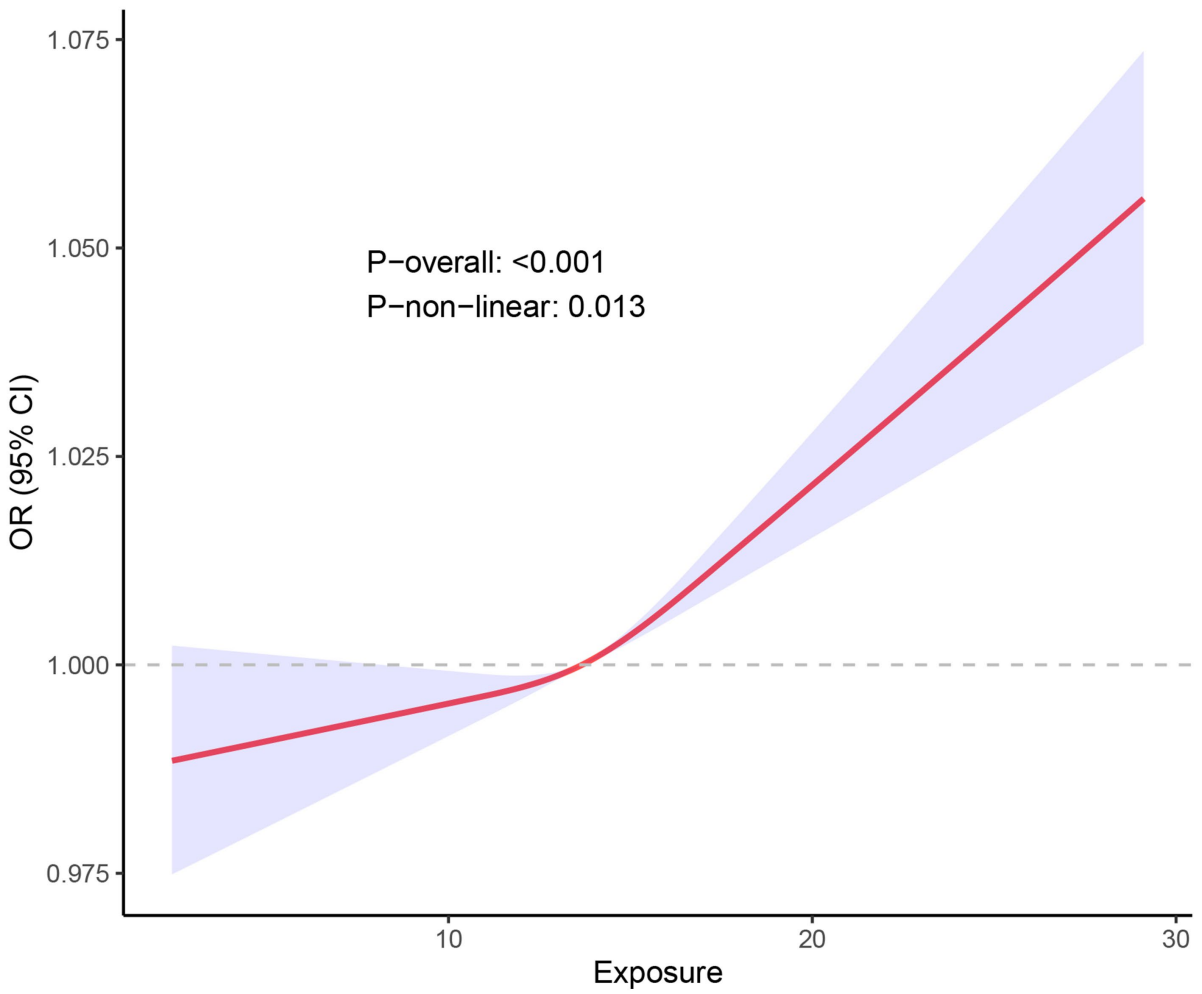
RCS fitting curve illustrated the association between NPAR and stroke incidence. X-axis (Exposure): Represents the range of NPAR values. Y-axis [OR (95% CI)]: Depicts the OR for stroke incidence along with its 95% CI. Red Curve Illustrates the trend in stroke incidence as the NPAR value increases. The shape of this curve indicates a complex, non-linear relationship rather than a simple linear correlation. Blue Shaded Area: Represents the 95% confidence interval for the red curve. A narrower shaded region indicates higher precision of the estimate, while a wider region indicates more uncertainty.

The RCS curve further illustrated the relationship between NPAR and stroke odds ratios (OR; Figure 3). The red solid line represents the estimated OR, while the shaded blue region indicates the 95% confidence interval (CI) around the OR. The overall relationship between NPAR and stroke was statistically significant (P-overall <0.001). Additionally, the analysis suggested a nonlinear association between NPAR and stroke prevalence (P-non-linear = 0.013). Low NPAR Values (<10): In this range, the OR remained close to 1, indicating no significant relationship between NPAR and stroke incidence. Medium NPAR Values (10–20): A slight upward trend was observed in the curve, suggesting a modest increase in stroke prevalence with rising NPAR values. High NPAR Values (>20): Above this threshold, the curve steepened significantly, accompanied by a wider CI, indicating a marked increase in stroke prevalence with higher NPAR values. Additionally, we calculated the Variance Inflation Factors (VIFs) for all covariates in the model, which ranged from 1.07 to 1.41. These values suggest that multicollinearity among the covariates is minimal, as VIFs below 2 are generally considered acceptable. Thus, multicollinearity was not a significant concern in our analysis.

### 3.3 Subgroup analysis

**Figure 4:**
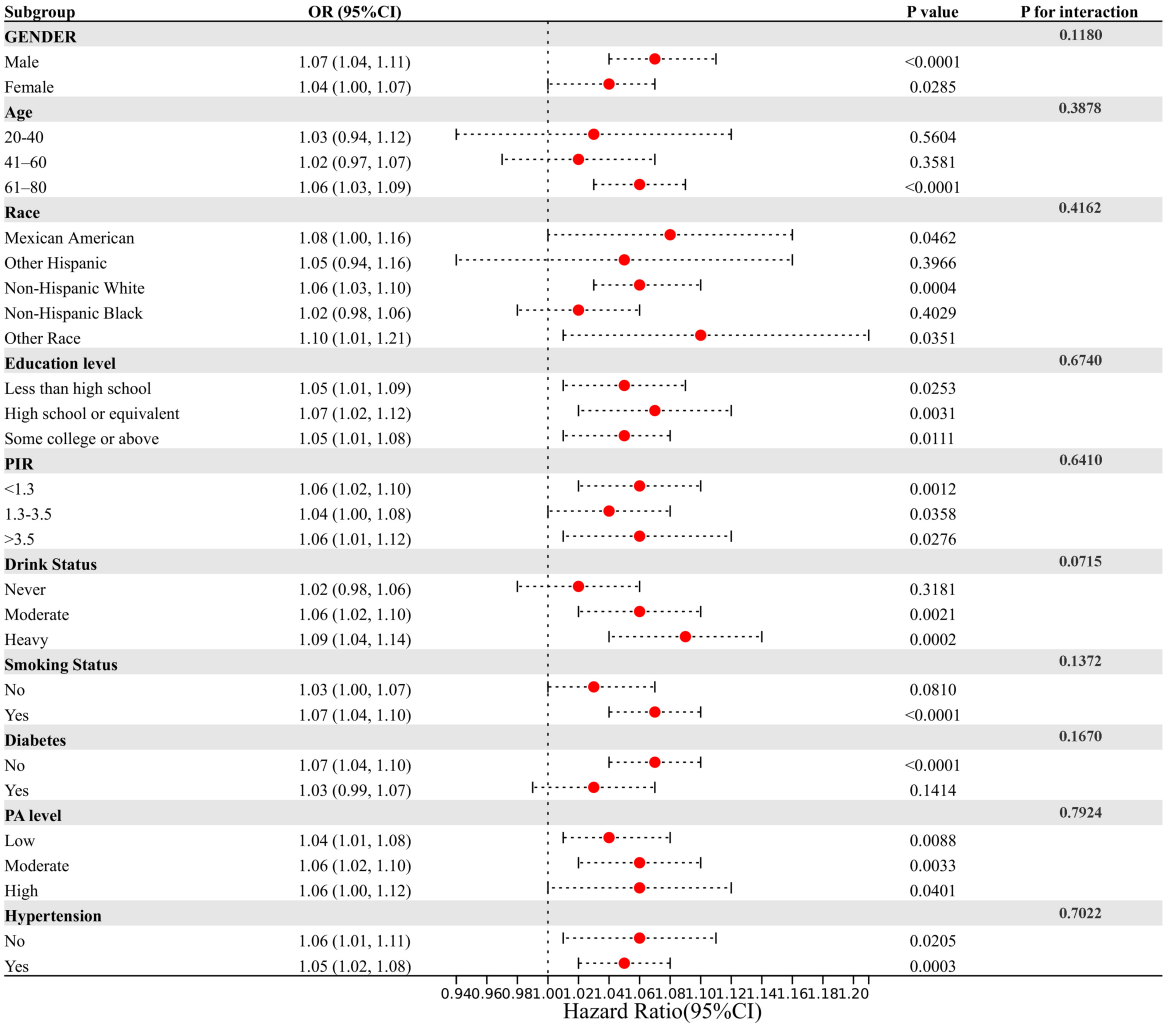
Assessment of the correlation between NPAR and stroke by subgroup analysis. This figure assesses the relationship between NPAR and stroke incidence across various demographic and clinical subgroups. Each row represents a specific subgroup, showing the OR and 95% CI for stroke incidence in relation to NPAR within that subgroup. The lack of significant interaction effects (P for interaction) in most subgroups suggests that the impact of NPAR on stroke prevalence is consistent across different demographic and clinical characteristics. However, certain subgroups—such as those defined by drinking status and age—may show slight differences in the NPAR-stroke relationship, though these interactions are not strongly significant.

Subgroup analyses and interaction testing were performed to examine whether the association between NPAR and stroke varied across different demographic groups. A forest plot was used to visualize these results (Figure 4). Our findings showed that the relationship between NPAR and stroke remained consistent across various subgroups, with all interaction *p*-values exceeding 0.05. This indicates that the association is independent of the stratifying factors, suggesting that NPAR may be a broadly applicable biomarker for stroke risk across diverse populations.

## 4 Discussion

Our study identified a significant association between stroke prevalence and NPAR in a large, community-representative cohort of 48,734 participants. Elevated NPAR levels were linked to higher stroke rates, and this positive correlation was consistently observed across various demographic subgroups. Subgroup analyses and interaction tests further supported the robustness of this association, suggesting that NPAR could serve as a valuable marker in stroke assessment. Notably, an inflection point was observed around an NPAR value of 14, indicating a threshold effect where stroke prevalence increases more sharply beyond this point. These findings underscore the potential importance of NPAR in stroke prevention and management, highlighting that elevated NPAR may be associated with an increased risk of stroke.

Research on stroke biomarkers has advanced to enhance the early detection of stroke prevalence. Following a literature review, we identified that stroke biomarkers mostly include the below categories: Lipid indicators, including TC and non-HDL, may more accurately predict stroke incidence than LDL (25); Inflammatory markers, especially IL-6, shown significant correlations with stroke risk (26); Hemodynamic indicators such as midregional proatrial natriuretic peptide and N-terminal pro-B-type natriuretic peptide shown promise in differentiating stroke subtypes (27, 28); Particular microRNAs (miR-125a-5p, miR-125b-5p, miR-143-3p) shown upregulation in acute ischemic stroke (29); Metabolomic investigations discovered new markers, including tetradecanedioate and hexadecanedioate, associated with cardioembolic stroke (30); Neurodegenerative indicators, such as total-tau and neurofilament light chain, were correlated with heightened stroke risk (31).

Neutrophil levels upon hospital admission have been associated with early neurological deterioration and poorer outcomes, especially in patients with intracerebral hemorrhage (ICH) (32). Albumin levels have been recognized as reliable biomarkers for predicting mortality and functional recovery in stroke patients (33, 34). There is a correlation between high albumin levels and improved functional recovery and decreased mortality in stroke patients, which suggests that some neuroprotective effects may be present (35). Albumin’s role as a major plasma antioxidant is crucial, contributing to delayed neuronal death phases (36), while reduced serum albumin can increase oxidative stress and lower antioxidant defenses. Inflammatory responses in ICH involve both neutrophils and albumin, and hypoalbuminemia, often seen in malnutrition, renal or hepatic issues, is linked to poorer health outcomes and greater frailty. Furthermore, albumin has been shown to independently predict pneumonia risk after acute ischemic stroke (37).

The combined use of neutrophil count and albumin levels has shown potential in improving the accuracy of stroke outcome predictions, integrating both acute inflammatory responses and nutritional status. Several studies have also highlighted the connection between NPAR and stroke-related outcomes. A 2021 retrospective study found that NPAR outperformed other biomarkers, such as albumin and neutrophil-to-leukocyte ratios, in predicting stroke-associated pneumoni (38). Chen et al. found that stroke patients with elevated NPAR had a higher risk of mortality (39), and Cui et al. reported worse outcomes in acute ICH patients with higher NPAR levels (40). Additionally, Lv and colleagues showed that increased NPAR was independently associated with adverse outcomes in both stroke-associated pneumonia and spontaneous ICH (41). These studies reinforce the strong link between NPAR and stroke, and our research provides further clarification of this relationship, suggesting that NPAR could serve as a novel biomarker for stroke risk assessment.

This is, to our knowledge, the first study to specifically investigate the relationship between NPAR and stroke prevalence. Previous research has primarily focused on NPAR’s association with cardiovascular diseases. For example, Zhang et al. found that high NPAR levels independently predicted coronary slow flow in patients with myocardial ischemia (42). Elevated NPAR levels in individuals with hypertension have also been associated with increased cardiovascular disease risk and overall mortality (43). Moreover, NPAR has been shown to correlate with coronary atherosclerosis in patients with chronic renal disease, accurately predicting the severity of the condition (44). These findings emphasize NPAR’s role as a marker of systemic inflammation and suggest that it may also reflect the severity of brain damage and post-stroke complications.

This study has notable strengths. The analysis was based on data from 48,734 U.S. adults, with sample weights adjusted to ensure representativeness of the broader U.S. population. However, several limitations must be acknowledged. As a cross-sectional study, we cannot establish a direct causal relationship between NPAR and stroke. Selection bias may also have influenced the results. To further explore the dynamics of NPAR in stroke progression, longitudinal studies with larger sample sizes are needed. Additionally, the absence of information on medication history and stroke subtype classification in the dataset introduces the potential for recall bias. Stroke diagnoses were based on self-reported questionnaires, which may further contribute to this bias. Future clinical investigations are necessary to validate these findings and explore the broader applicability of NPAR in stroke detection and management.

## 5 Conclusion

Our study demonstrates that elevated NPAR levels are associated with increased stroke prevalence. This finding offers new insights for primary stroke prevention, suggesting that NPAR could serve as a practical tool for assessing stroke likelihood. By incorporating NPAR into clinical decision-making, healthcare providers could implement timely preventive measures for individuals with higher NPAR levels, ultimately optimizing stroke prevention strategies and improving patient outcomes.

## Data Availability

The original contributions presented in the study are included in the article/supplementary material, further inquiries can be directed to the corresponding authors.

## References

[1] Kuriakose D Xiao Z. Pathophysiology and treatment of stroke: present status and future perspectives. Int J Mol Sci. (2020) 21. doi: 10.3390/ijms21207609, PMID: 33076218 PMC7589849

[2] GBD 2019 Stroke Collaborators. Global, regional, and national burden of stroke and its risk factors, 1990-2019: a systematic analysis for the global burden of disease study 2019. Lancet Neurol. (2021) 20:795–820. doi: 10.1016/S1474-4422(21)00252-0 34487721 PMC8443449

[3] Roth GA Mensah GA Johnson CO Addolorato G Ammirati E Baddour LM. Global burden of cardiovascular diseases and risk factors, 1990-2019: update from the GBD 2019 study. J Am Coll Cardiol. (2020) 76:2982–3021. doi: 10.1016/j.jacc.2020.11.010 33309175 PMC7755038

[4] Feigin VL Brainin M Norrving B Martins S Sacco RL Hacke W. World stroke organization (WSO): global stroke fact sheet 2022. Int J Stroke. (2022) 17:18–29. doi: 10.1177/17474930211065917, PMID: 34986727

[5] Owolabi MO Thrift AG Mahal A Ishida M Martins S Johnson WD. Primary stroke prevention worldwide: translating evidence into action. Lancet Public Health. (2022) 7:e74–85. doi: 10.1016/S2468-2667(21)00230-9, PMID: 34756176 PMC8727355

[6] Cai X Hu J Wen W Wang M Zhu Q Liu S. Association between the geriatric nutritional risk index and the risk of stroke in elderly patients with hypertension: a longitudinal and cohort study. Front Nutr. (2022) 9:1048206. doi: 10.3389/fnut.2022.1048206, PMID: 36562034 PMC9763600

[7] Cai X Hu J Zhu Q Wang M Liu S Dang Y. Relationship of the metabolic score for insulin resistance and the risk of stroke in patients with hypertension: a cohort study. Front Endocrinol (Lausanne). (2022) 13:1049211. doi: 10.3389/fendo.2022.1049211, PMID: 36545329 PMC9760826

[8] Anrather J Iadecola C. Inflammation and stroke: an overview. Neurotherapeutics. (2016) 13:661–70. doi: 10.1007/s13311-016-0483-x, PMID: 27730544 PMC5081118

[9] Chamorro Á Dirnagl U Urra X Planas AM. Neuroprotection in acute stroke: targeting excitotoxicity, oxidative and nitrosative stress, and inflammation. Lancet Neurol. (2016) 15:869–81. doi: 10.1016/S1474-4422(16)00114-9 27180033

[10] Danton GH Dietrich WD. Inflammatory mechanisms after ischemia and stroke. J Neuropathol Exp Neurol. (2003) 62:127–36. doi: 10.1093/jnen/62.2.127, PMID: 12578222

[11] Mortaz E Alipoor SD Adcock IM Mumby S Koenderman L. Update on neutrophil function in severe inflammation. Front Immunol. (2018) 9:2171. doi: 10.3389/fimmu.2018.02171 30356867 PMC6190891

[12] Strecker JK Schmidt A Schäbitz WR Minnerup J. Neutrophil granulocytes in cerebral ischemia - evolution from killers to key players. Neurochem Int. (2017) 107:117–26. doi: 10.1016/j.neuint.2016.11.006 27884770

[13] Arques S. Human serum albumin in cardiovascular diseases. Eur J Intern Med. (2018) 52:8–12. doi: 10.1016/j.ejim.2018.04.014, PMID: 29680174

[14] Roche M Rondeau P Singh NR Tarnus E Bourdon E. The antioxidant properties of serum albumin. FEBS Lett. (2008) 582:1783–7. doi: 10.1016/j.febslet.2008.04.057, PMID: 18474236

[15] He X Dai F Zhang X Pan J. The neutrophil percentage-to-albumin ratio is related to the occurrence of diabetic retinopathy. J Clin Lab Anal. (2022) 36:e24334. doi: 10.1002/jcla.24334, PMID: 35285099 PMC8993596

[16] Famakin B Weiss P Hertzberg V McClellan W Presley R Krompf K. Hypoalbuminemia predicts acute stroke mortality: Paul Coverdell Georgia stroke registry. J Stroke Cerebrovasc Dis. (2010) 19:17–22. doi: 10.1016/j.jstrokecerebrovasdis.2009.01.015, PMID: 20123222

[17] Kang L Yu H Yang X Zhu Y Bai X Wang R. Neutrophil extracellular traps released by neutrophils impair revascularization and vascular remodeling after stroke. Nat Commun. (2020) 11:2488. doi: 10.1038/s41467-020-16191-y, PMID: 32427863 PMC7237502

[18] Cui H Ding X Li W Chen H Li H. The neutrophil percentage to albumin ratio as a new predictor of in-hospital mortality in patients with ST-segment elevation myocardial infarction. Med Sci Monit. (2019) 25:7845–52. doi: 10.12659/MSM.917987, PMID: 31628741 PMC6820334

[19] Yang D Niu C Li P Du X Zhao M Jing W. Study of the neutrophil percentage-to-albumin ratio as a biomarker for predicting recurrence of first-episode ischemic stroke. J Stroke Cerebrovasc Dis. (2024) 33:107485. doi: 10.1016/j.jstrokecerebrovasdis.2023.107485, PMID: 37966092

[20] Zawiah M Khan AH Farha RA Usman A Al-Ashwal FY Akkaif MA. Assessing the predictive value of neutrophil percentage to albumin ratio for ICU admission in ischemic stroke patients. Front Neurol. (2024) 15:1322971. doi: 10.3389/fneur.2024.1322971, PMID: 38361641 PMC10868651

[21] Curtin LR Mohadjer LK Dohrmann SM Kruszon-Moran D Mirel LB Carroll MD. National Health and nutrition examination survey: sample design, 2007-2010. Vital Health Stat. (2013) 2:1–23. 25090039

[22] Xie R Zhang Y. Association between 19 dietary fatty acids intake and rheumatoid arthritis: results of a nationwide survey. Prostaglandins Leukot Essent Fat Acids. (2023) 188:102530. doi: 10.1016/j.plefa.2022.102530, PMID: 36586398

[23] Liu CF Chien LW. Predictive role of neutrophil-percentage-to-albumin ratio (NPAR) in nonalcoholic fatty liver disease and advanced liver fibrosis in nondiabetic US adults: evidence from NHANES 2017-2018. Nutrients. (2023) 15:1892. doi: 10.3390/nu15081892 37111111 PMC10141547

[24] Yang L Chen X Cheng H Zhang L. Dietary copper intake and risk of stroke in adults: a case-control study based on National Health and nutrition examination survey 2013-2018. Nutrients. (2022) 14:409. doi: 10.3390/nu14030409 35276768 PMC8839334

[25] Harshfield EL Markus HS. Association of Baseline Metabolomic Profiles with Incident Stroke and Dementia and with imaging markers of cerebral small vessel disease. Neurology. (2023) 101:e489–501. doi: 10.1212/WNL.0000000000207458, PMID: 37290969 PMC10401678

[26] Jenny NS Callas PW Judd SE McClure LA Kissela B Zakai NA. Inflammatory cytokines and ischemic stroke risk: the REGARDS cohort. Neurology. (2019) 92:e2375–84. doi: 10.1212/WNL.0000000000007416 31004072 PMC6598817

[27] Bustamante A López-Cancio E Pich S Penalba A Giralt D García-Berrocoso T. Blood biomarkers for the early diagnosis of stroke: the stroke-Chip study. Stroke. (2017) 48:2419–25. doi: 10.1161/STROKEAHA.117.017076, PMID: 28716979

[28] Katan M Moon YP Paik MC Mueller B Huber A Sacco RL. Procalcitonin and Midregional Proatrial natriuretic peptide as markers of ischemic stroke: the northern Manhattan study. Stroke. (2016) 47:1714–9. doi: 10.1161/STROKEAHA.115.011392, PMID: 27197849 PMC4927365

[29] Tiedt S Prestel M Malik R Schieferdecker N Duering M Kautzky V. RNA-Seq identifies circulating miR-125a-5p, miR-125b-5p, and miR-143-3p as potential biomarkers for acute ischemic stroke. Circ Res. (2017) 121:970–80. doi: 10.1161/CIRCRESAHA.117.311572, PMID: 28724745

[30] Sun D Tiedt S Yu B Jian X Gottesman RF Mosley TH. A prospective study of serum metabolites and risk of ischemic stroke. Neurology. (2019) 92:e1890–8. doi: 10.1212/WNL.0000000000007279, PMID: 30867269 PMC6550501

[31] Heshmatollah A Fani L Koudstaal PJ Ghanbari M Ikram MA Ikram MK. Plasma β-amyloid, Total-tau, and Neurofilament light chain levels and the risk of stroke: a prospective population-based study. Neurology. (2022) 98:e1729–37. doi: 10.1212/WNL.0000000000200004, PMID: 35232820

[32] Lattanzi S Cagnetti C Provinciali L Silvestrini M. Neutrophil-to-lymphocyte ratio predicts the outcome of acute intracerebral hemorrhage. Stroke. (2016) 47:1654–7. doi: 10.1161/STROKEAHA.116.013627, PMID: 27165957

[33] Artero A Zaragoza R Camarena JJ Sancho S González R Nogueira JM. Prognostic factors of mortality in patients with community-acquired bloodstream infection with severe sepsis and septic shock. J Crit Care. (2010) 25:276–81. doi: 10.1016/j.jcrc.2009.12.004, PMID: 20149587

[34] Park JE Chung KS Song JH Kim SY Kim EY Jung JY. The C-reactive protein/albumin ratio as a predictor of mortality in critically ill patients. J Clin Med. (2018) 7:333. doi: 10.3390/jcm7100333, PMID: 30297655 PMC6210319

[35] Idicula TT Waje-Andreassen U Brogger J Naess H Thomassen L. Serum albumin in ischemic stroke patients: the higher the better. The Bergen Stroke Study Cerebrovasc Dis. (2009) 28:13–7. doi: 10.1159/000215938, PMID: 19420917

[36] Halliwell B. Albumin—An important extracellular antioxidant? Biochem Pharmacol. (1988) 37:569–71. doi: 10.1016/0006-2952(88)90126-8, PMID: 3277637

[37] Dziedzic T Pera J Klimkowicz A Turaj W Slowik A Rog TM. Serum albumin level and nosocomial pneumonia in stroke patients. Eur J Neurol. (2006) 13:299–301. doi: 10.1111/j.1468-1331.2006.01210.x, PMID: 16618350

[38] Zhang H Wu T Tian X Lyu P Wang J Cao Y. High neutrophil percentage-to-albumin ratio can predict occurrence of stroke-associated infection. Front Neurol. (2021) 12:705790. doi: 10.3389/fneur.2021.705790, PMID: 34566849 PMC8455847

[39] Chen Z Xie D Li Y Dai Z Xiang S Chen Z. Neutrophil albumin ratio is associated with all-cause mortality in stroke patients: a retrospective database study. Int J Gen Med. (2022) 15:1–9. doi: 10.2147/IJGM.S323114, PMID: 35018109 PMC8742575

[40] Cui T Wang C Zhu Q Li S Yang Y Wang A. Association between neutrophil percentage-to-albumin ratio and 3-month functional outcome in acute ischemic stroke patients with reperfusion therapy. Front Neurol. (2022) 13:898226. doi: 10.3389/fneur.2022.898226, PMID: 36176549 PMC9513151

[41] Lv XN Shen YQ Li ZQ Deng L Wang ZJ Cheng J. Neutrophil percentage to albumin ratio is associated with stroke-associated pneumonia and poor outcome in patients with spontaneous intracerebral hemorrhage. Front Immunol. (2023) 14:1173718. doi: 10.3389/fimmu.2023.1173718, PMID: 37388726 PMC10300413

[42] Zang SW Long JJ Wang Y. Neutrophil percentage to albumin ratio as a predictor for coronary slow flow phenomenon in patients with myocardial ischemia with no obstructive coronary arteries. Int J Gen Med. (2024) 17:3511–9. doi: 10.2147/IJGM.S477431 39161405 PMC11330862

[43] Liu Z Dong L Shen G Sun Y Liu Y Mei J. Associations of neutrophil-percentage-to-albumin ratio level with all-cause mortality and cardiovascular disease-cause mortality among patients with hypertension: evidence from NHANES 1999-2010. Front Cardiovasc Med. (2024) 11:1397422. doi: 10.3389/fcvm.2024.1397422 39087072 PMC11288876

[44] Zhao M Huang X Zhang Y Wang Z Zhang S Peng J. Predictive value of the neutrophil percentage-to-albumin ratio for coronary atherosclerosis severity in patients with CKD. BMC Cardiovasc Disord. (2024) 24:277. doi: 10.1186/s12872-024-03896-x, PMID: 38807036 PMC11134736

